# Heterostructure Ni_3_S_4_–MoS_2_ with interfacial electron redistribution used for enhancing hydrogen evolution†

**DOI:** 10.1039/d1ra02828f

**Published:** 2021-06-01

**Authors:** Jingmin Ge, Jiaxing Jin, Yanming Cao, Meihong Jiang, Fazhi Zhang, Hongling Guo, Xiaodong Lei

**Affiliations:** State Key Laboratory of Chemical Resource Engineering, Beijing University of Chemical Technology Beijing 100029 China leixd@mail.buct.edu.cn +86-10-64455357; Institute of Forensic Science, Ministry of Public Security Beijing 100038 China guohongling1234@163.com

## Abstract

Developing highly effective and inexpensive electrocatalysts for hydrogen evolution reaction (HER), particularly in a water-alkaline electrolyzer, are crucial to large-scale industrialization. The earth-abundant molybdenum disulfide (MoS_2_) is an ideal electrocatalyst in acidic media but suffers from a high overpotential in alkaline solution. Herein, nanospherical heterostructure Ni_3_S_4_–MoS_2_ was obtained *via* a one-pot synthesis method, in which Ni_3_S_4_ was uniformly integrated with MoS_2_ ultrathin nanosheets. There were abundant heterojunctions in the as-synthesized catalyst, which were verified by X-ray photoelectron spectroscopy (XPS) and high-resolution transmission electron microscopy (HRTEM). The structure features with interfacial electron redistribution was proved by XPS and density functional theory (DFT) calculations, which offered several advantages to promote the HER activity of MoS_2_, including increased specific surface area, exposed abundant active edge sites and improved electron transfer. Ni_3_S_4_–MoS_2_ exhibited a low overpotential of 116 mV at 10 mA cm^−2^ in an alkaline solution with a corresponding Tafel slope of 81 mV dec^−1^ and long-term stability of over 20 h. DFT simulations indicated that the synergistic effects in the system with the chemisorption of H on the (002) plane of MoS_2_ and OH on the (311) plane of Ni_3_S_4_ accelerated the rate-determining water dissociation steps of HER. This study provides a valuable route for the design and synthesis of inexpensive and efficient HER electrocatalyst, heterostructure Ni_3_S_4_–MoS_2_.

## Abstract 2

The heterostructure of Ni_3_S_4_–MoS_2_ as an efficient hydrogen evolution catalyst was fabricated by a one-pot hydrothermal method. The as-synthesized catalyst with interfacial electron redistribution generated numerous active sites.

## Graphical abstract

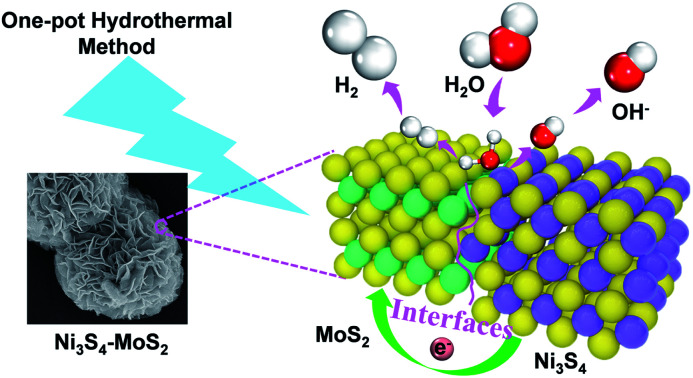

## Introduction

With the increasing environmental protection demands, developing sustainable and fossil-free renewable energy plays a major role.^1^ Due to its environmentally friendly, zero-emission, high-energy capacity and sustainable merits, hydrogen (H_2_) has received extensive attention.^2^ The most efficient method to generate H_2_ (2H_2_O → 2H_2_ + O_2_) is water splitting by electric power generated from renewable energy sources.^3–5^ Currently, some noble metals, such as Pt, Rh and Ir, have been proved to possess excellent catalytic performance as HER electrocatalysts, including low overpotential, low Tafel slope and low impedance.^6,7^ However, the exorbitant cost and limited earth abundance of these noble metal materials have hindered their industrialization and commercialization.^8^ Therefore, developing electrocatalysts that are low cost, highly active and stable from nonprecious and earth-abundant metal materials is urgent.

In recent years, earth-abundant 2D MoS_2_ has been considered as an ideal alternative to the precious Pt-based catalysts as the next-generation electrocatalytic material due to its unique structure and chemical properties.^9–11^ DFT calculations indicated that MoS_2_ exhibits excellent HER performance in acidic solutions since its edge sites permit a near-optimal hydrogen adsorption free energy (Δ*G*_H*_ = 0.08 eV).^12^ Moreover, the tremendous amount of S sites in the basal plane of pure MoS_2_ are quite inert and not sufficiently utilized.^13^ Unfortunately, MoS_2_ has also been found to have poor activity in alkaline media,^14^ even though alkaline catalysis is a more widespread application. Numerous strategies have been employed to improve the catalytic activity of 2D MoS_2_, such as generating the sulfur vacancies,^15^ introducing heteroatoms,^16^ changing conductive supports.^17^ It has been both experimentally and theoretically identified that the fabrication of heterogeneous nanostructures with abundant and accessible exposed active sites is a very effective way for improving the catalytic activity.^18^

MoS_2_ decorated with transition metals, such as Fe, Co and Ni, by heterostructure engineering has shown excellent electrocatalytic performance.^19^ Because of the versatile electronic structure of these metals and the ability to fill d orbitals with electrons from another transition metal, they provide distinctive catalytic properties. For example, nickel-based catalysts have been shown to have impressive potential for HER electrocatalysts due to their conductivity and low cost.^20,21^ Some reports have indicated that constructing heterostructure for MoS_2_*via* introducing nickel sulfides (*e.g.*, Ni_3_S_2_,^22^ NiS_2_ (ref. 23) and NiS^24^) could provide superior HER activity. However, the intrinsic motivation of the enhanced catalytic performance is not clear. Moreover, the impact of constructing a heterogeneous structure *via* introducing Ni_3_S_4_ into MoS_2_ on HER performance remains to be studied. Moreover, an in-depth understanding of the interfacial electron redistribution for the improved HER performance is important for its wide application.

In this study, we fabricated the heterostructure Ni_3_S_4_–MoS_2_ with a nanospherical morphology *via* a one-step hydrothermal strategy and applied it as an HER catalyst. To improve the electronic conductivity and expose abundant active edge sites of MoS_2_, we constructed the heterostructure *via* introducing Ni_3_S_4_ into MoS_2_ ultra-thin nanosheets, which significantly enhanced the HER activity. The heterostructure of Ni_3_S_4_–MoS_2_ was investigated *via* XPS, SEM and HRTEM techniques. For researching the electron redistribution on the interface of Ni_3_S_4_–MoS_2_ and the mechanism of electro-catalysis during HER, we also applied the DFT simulation. It is indicated that the heterostructure Ni_3_S_4_–MoS_2_ optimized water dissociation energies and H* absorption free energy.

## Experimental section

### Materials

Sodium molybdate (vi) dihydrate (Na_2_MoO_4_·2H_2_O), nickel(ii) chloride hexahydrate (NiCl_2_·6H_2_O), urea, sodium sulfide nonahydrate (Na_2_S·9H_2_O) and thiourea (CH_4_N_2_S) were obtained from Beijing Chemical Works. A 5 wt% Nafion solution was obtained from Du Pont China Holding Co., Ltd. A nickel foam (NF) having a thickness of 0.5 mm was bought from Changsha Lyrun New Materials Co., Ltd., and it (cut as 1.0 cm × 1.0 cm in size) was washed with hydrochloric acid, ethanol and deionized water. All other chemicals were of analytical grade and were purchased from Sinopharm Chemical Reagents Co. All chemicals were used without further purification.

### Synthesis of MoS_2_, Ni_4_S_3_–MoS_2_ and Ni_4_S_3_ nanosheets

3.5 mmol (0.847 g) of Na_2_MoO_4_·2H_2_O and 15 mmol (1.14 g) of thiourea were dissolved in 30 mL of deionized water and dispersed by ultrasonication for 10 min to form a uniform solution. The mixture was transferred into a 50 mL Teflon-lined stainless-steel autoclave and maintained at 180 °C for 24 h. The as-prepared MoS_2_ was washed with deionized water and ethanol several times and then dried in a vacuum at 60 °C. Ni_3_S_4_–MoS_2_ was synthesized by a simple one-pot step method that was the same as the above process for MoS_2_. However, in the Na_2_MoO_4_·2H_2_O and thiourea mixed solution, different amounts of NiCl_2_·6H_2_O were added. Then, the Ni_3_S_4_–MoS_2_ nanosheets were obtained after the same hydrothermal treatment, washing and drying.

Ni_3_S_4_ was prepared according to the method in the reported literature.^25^ Briefly, 1.5 mmol (0.357 g) of NiCl_2_·6H_2_O and 10 mmol (0.60 g) of urea were dissolved in 30 mL deionized water, and the mixture was dispersed by ultrasonication for 10 min to form a uniform solution. The solution was then transferred into a 50 mL Teflon-lined stainless-steel autoclave and maintained at 130 °C for 2 h. The as-prepared Ni(OH)_2_ was washed with deionized water and ethanol several times and then dried in a vacuum at 60 °C. Then, the as-prepared Ni(OH)_2_ precursor together with 30 mL of 15 mmol (3.6 g) sodium sulfide (Na_2_S·9H_2_O) aqueous solution was placed in the Teflon-lined stainless-steel autoclave and maintained at 90 °C for 9 h. The as-prepared Ni_3_S_4_ was washed with deionized water and ethanol several times and then dried in a vacuum at 60 °C for 12 h.

### Characterization

X-ray diffraction (XRD) patterns were collected on a Rigaku XRD-6000 diffractometer using Cu Kα radiation from 3° to 80° at the scan rate of 10° min^−1^. The morphologies were investigated *via* SEM (Zeiss SUPRA 55) at an accelerating voltage of 20 kV. A Brunauer–Emmett–Teller (BET, ASAP 2460) apparatus was used to measure the surface area. HRTEM images were recorded using a JEOL JEM-2010 field-emission transmission electron microscope at an accelerating voltage of 200 kV, combined with energy-dispersive X-ray spectroscopy (EDS). XPS measurements were performed on a Thermo VG ESCALAB 250 X-ray photoelectron spectrometer with Al Kα radiation at a pressure of about 2 × 10^−9^ Pa. Inductively coupled plasma-optical emission spectrometry (ICP-OES) was adopted to analyze the chemical components of the catalysts.

### Electrochemical measurements

Electrochemical measurements were performed on an electrochemical workstation (CHI 660E, CH Instruments Inc., Chenhua, Shanghai) using a three-electrode mode in an Ar-saturated 1 mol L^−1^ KOH aqueous solution. A platinum electrode was used as the counter electrode, a sliver/sliver chloride (Ag/AgCl) electrode was used as the reference electrode, and the as-fabricated materials were used as the working electrodes. The potentials were converted to the RHE scale using the following Nernst equation: (*E*(RHE) = *E*(Ag/AgCl) + 0.059 pH + 0.197). To accelerate the electrochemical performance tests, 5 mg of the as-prepared catalysts, 2 mg conductive carbon, 35 μL of the 5 wt% Nafion solution and 1 mL anhydrous ethanol were mixed and ultrasonicated for 10 min to form homogeneous catalyst inks. The catalyst inks were dripped respectively onto the as-prepared NF to obtain the working electrodes with a loading of ∼5 mg cm^−2^, which were dried at 60 °C for 1 h. The electrochemical impedance spectroscopy (EIS) tests were performed in the frequency range from 100 kHz to 0.1 Hz at an overpotential of 180 mV. The cyclic voltammograms (CV) were obtained between 0.1 and −0.3 V *vs.* RHE at 100 mV s^−1^ to investigate the cycling stability. The long-term stability tests were recorded by taking a chronoamperometric curve current density that reached 10 mA cm^−2^. All data were presented without IR compensation, and all the electrochemical tests were tested at room temperature.

### Computational methods

All first principles calculations were performed *via* DFT in the Cambridge Sequential Total Energy Package (CASTEP) module in the Materials Studio. The MoS_2_ (002) plane consisting of six layers of Mo and S, and the Ni_3_S_4_ (311) slab composed of six layers of atoms were constructed as our models, because the (002) plane of MoS_2_ and the (311) plane of Ni_3_S_4_ were dominant crystal faces from the HRTEM images. The exchange–correlation interactions were treated within the generalized gradient approximation of the Perdew–Burke–Ernzerhof (PBE) type. The plane-wave cutoff energy was 400 eV, and a k-mesh of 3 × 3 × 1 was adopted to sample the Brillouin zone. The convergence threshold for energy and Hellmann–Feynman forces on each atom were set to 10^−5^ eV and 0.01 eV Å^−1^. Vacuum layers of 15 Å were introduced to minimize interactions between adjacent layers in all supercells.^15^ All the atom positions in the model were optimized by the conjugate-gradient optimization procedure.

Gibbs free-energy of the adsorption atomic hydrogen was calculated as the following formula:

1 Δ*G* = Δ*E* + Δ*ZPE* − *T*Δ*S*

where Δ*E* is the adsorption energy of adsorbed species on the given unit cell. Δ*ZPE* and *T*Δ*S* are the zero-point energy and entropy difference of hydrogen in the adsorbed state and the gas phase, respectively. The value of *ZPE* and *TS* for the adsorbed species were calculated from the vibration frequencies, as shown in the previous literature.^15^

## Results and discussion

### Morphology and structure of Ni_3_S_4_–MoS_2_

**Fig. 1:**
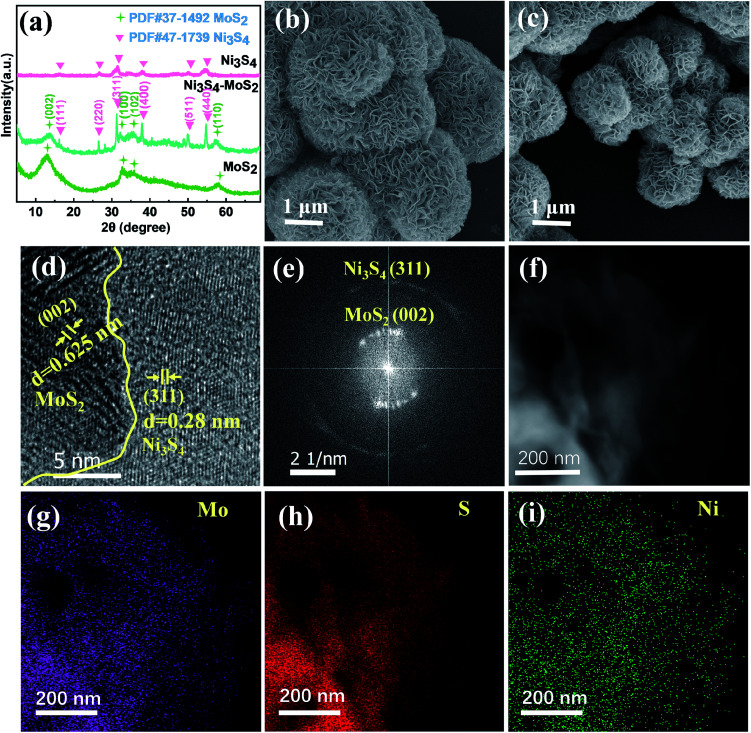
(a) XRD patterns of Ni_3_S_4_–MoS_2_, Ni_3_S_4_ and MoS_2_. (b) SEM image of the Ni_3_S_4_–MoS_2_. (c) SEM image of the MoS_2_. (d) TEM image and (e) corresponding SAED pattern of Ni_3_S_4_–MoS_2_. (f) TEM image of Ni_3_S_4_–MoS_2_ and corresponding elemental mappings: Mo (g), S (h) and Ni (i).

A series of Ni_3_S_4_–MoS_2_ with different Ni contents were synthesized *via* a one-pot hydrothermal method. The ICP-OES measurements provided the contents of Ni in Ni_3_S_4_–MoS_2_, as shown in Table S1.† The XRD patterns of the Ni_3_S_4_–MoS_2_ composite, pure Ni_3_S_4_ and MoS_2_ are shown in Fig. 1a. For the Ni_3_S_4_ and MoS_2_ samples, all of their peaks were well matching with the Ni_3_S_4_ (JCPDS no. 47-1739) and MoS_2_ phases (JCPDS no. 37-1492), respectively. For the XRD pattern of Ni_3_S_4_–MoS_2_, the diffraction peaks at 14.5°, 32.7° and 58.3° corresponded to the (002), (100) and (110) lattice planes of the MoS_2_ phase (JCPDS Card no. 37-1492).^26^ There are six sharp peaks at 16.2°, 26.6°, 31.3°, 37.9°, 50.0° and 54.7°, which can be indexed to the (111), (220), (311), (400), (511) and (440) planes of Ni_3_S_4_ (JCPDS no. 47-1739).^27^ The XRD diagram implied the hybrid of these two metal sulfides in Ni_3_S_4_–MoS_2_.

The SEM image of Ni_3_S_4_–MoS_2_ identifies that the morphology of the sample is a hierarchical nanosphere composed of nanosheets with a size of about 1 μm and a thickness of about 30 nm (Fig. 1b). By comparison Fig. 1b with c, it is found that the nanosheets of Ni_3_S_4_–MoS_2_ are bigger and thinner than that of MoS_2_ (average size is about 0.6 μm and thickness is 40 nm, as shown in Fig. S1†). Besides, the heterostructure Ni_3_S_4_–MoS_2_ has a larger specific surface area (2.7 m^2^ g^−1^) than that of the pure MoS_2_ (0.6 m^2^ g^−1^). The information illustrates that the construction of the heterojunction increased the surface area of samples. The HRTEM images provide further details of the microstructure for the heterostructure Ni_3_S_4_–MoS_2_. The lattice spacing of 0.625 nm corresponds to the (002) plane of MoS_2_,^28,29^ and 0.28 nm corresponds to the (311) plane of Ni_3_S_4_,^30^ indicating that the sample consisted of Ni_3_S_4_ and MoS_2_. The HRTEM image suggests that the (002) plane of MoS_2_ and (311) plane of Ni_3_S_4_ constitute important heterointerfaces in the composite (Fig. 1d). The crystal structure of the Ni_3_S_4_–MoS_2_ composite was further verified by the select area electron diffraction (SAED) (Fig. 1e). It showed that the inner ring was strong and the outer ring pattern correspond to the (002) plane of the MoS_2_ crystal and the (311) plane of Ni_3_S_4_, respectively, matching well with the XRD spectra (Fig. 1a). The EDS elemental mapping of Ni_3_S_4_–MoS_2_ indicates that the Mo, S and Ni elements distributed uniformly in the entire nanosheets (Fig. 1f–i), and the contents of Mo, S and Ni of Ni_3_S_4_–MoS_2_ are consistent with the results of ICP-OES tests (Fig. S2 and Table S2†).

**Fig. 2:**
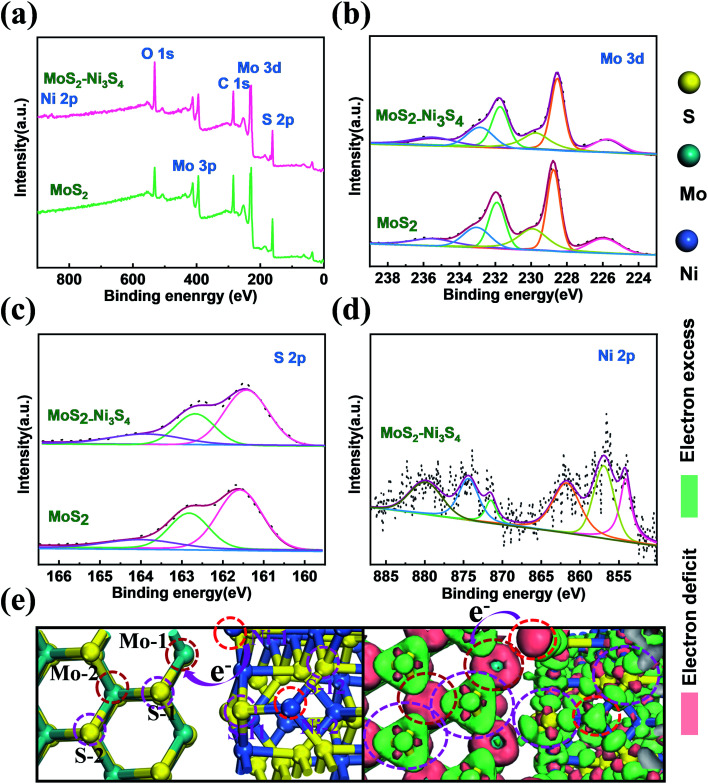
XPS spectra of Ni_3_S_4_–MoS_2_ and MoS_2_: (a) Survey spectra, (b) Mo 3d, (c) S 2p and (d) Ni 2p. (e) Top views of the most stable structure and charge density difference for the Ni_3_S_4_–MoS_2_.

XPS spectra were obtained to confirm the chemical composition and valence state of atoms in Ni_3_S_4_–MoS_2_ and pure MoS_2_, as shown in Fig. 2. It shows the existence of C, Mo, S, Ni and O elements in Ni_3_S_4_–MoS_2_ and the presence of all the elements but no Ni signal in MoS_2_ (Fig. 2a). The O 1s signal was assigned to the absorbed O-containing species, such as O_2_, CO_2_ and H_2_O. The high-resolution Mo 3d spectra of Ni_3_S_4_–MoS_2_ directly evidence the concurrent presence of Mo^3+^ and Mo^4+^ (Fig. 2b) with four definite fitting peaks assigned to Mo^3+^ (3d_3/2_ at 231.8 eV and 3d_5/2_ at 228.6 eV) and Mo^4+^ (3d_3/2_ at 232.8 eV and 3d_5/2_ at 229.7 eV), respectively.^17^ The binding energies of Mo 3d_5/2_ and Mo 3d_3/2_ in Ni_3_S_4_–MoS_2_ are negative shifts (0.2 eV) compared with the corresponding peaks of pristine MoS_2_,^31,32^ indicating the lower valence state of Mo in Ni_3_S_4_–MoS_2_. Thus, the charge transfer behavior from Ni_3_S_4_ to MoS_2_ is determined. The change of Mo valence was ascribed to the electronegativity difference between Mo and Ni, where the introduced Ni_3_S_4_ led to the rearrangement of the electron cloud between Mo and S, thus, forming a new hybridized electronic state. The S 2p spectrum of Ni_3_S_4_–MoS_2_ (Fig. 2c) was deconvoluted into three peaks at 161.4 (S 2p_3/2_), 162.6 (S 2p_1/2_) and 164.8 eV (S^2−^), respectively, and the S 2p shifts to the lower binding energy (about 0.2 eV, S 2p_3/2_ and S 2p_3/2_) compared with the pristine MoS_2_ (Fig. 2c), indicating the lower valence state of S in Ni_3_S_4_–MoS_2_. The result was consistent with the previous reports for other transition metals decorated with MoS_2_.^33,34^ The lower valence state of S in Ni_3_S_4_–MoS_2_ than that in MoS_2_ indicated that S in Ni_3_S_4_–MoS_2_ had a higher electric charge density that was contributed to H adsorption, which helped to improve the HER properties. The Ni 2p spectrum of Ni_3_S_4_–MoS_2_, as shown in Fig. 2d, exhibits the apparent Ni 2p_3/2_ and 2p_1/2_ peaks at 854.8 and 872.1 eV, respectively, that are attributed to Ni^2+^, and the other peaks at 856.9 and 874.3 eV, attributed to Ni^3+^.^21^ Two satellite peaks at 862.1 and 879.6 eV attributed to Ni 2p_3/2_ and 2p_1/2_, respectively, were observed.^35^ It was further confirmed that the material contained compound Ni_3_S_4_,^36^ which is in agreement with the XRD and HRTEM measurements.

To investigate the electron redistribution on the heterostructure of Ni_3_S_4_–MoS_2_, the electron density different diagram and the Bader charge analysis was performed by DFT calculations (Fig. 2e and Table S3†). The different electron density diagrams demonstrated that the electric charge densities of Mo and S increased on the interfaces Mo-1, S-1 and S-11 in Ni_3_S_4_–MoS_2_, compared with that on the base Mo-2, S-2 and S-12 in Ni_3_S_4_–MoS_2_, indicating that the binding energies of Mo and S in the heterojunction of Ni_3_S_4_–MoS_2_ reduced, which is in accordance with the XPS spectra and Bader charge analysis. The electric charge density of Ni on the interface Ni-1, compared with that on the base Ni-2 in Ni_3_S_4_–MoS_2_, was decreased, indicating that the electrons were transferred from Ni to Mo or S on the interfaces. These data confirmed the electron redistribution in the heterostructure Ni_3_S_4_–MoS_2_.

### HER catalytic behavior

**Fig. 3:**
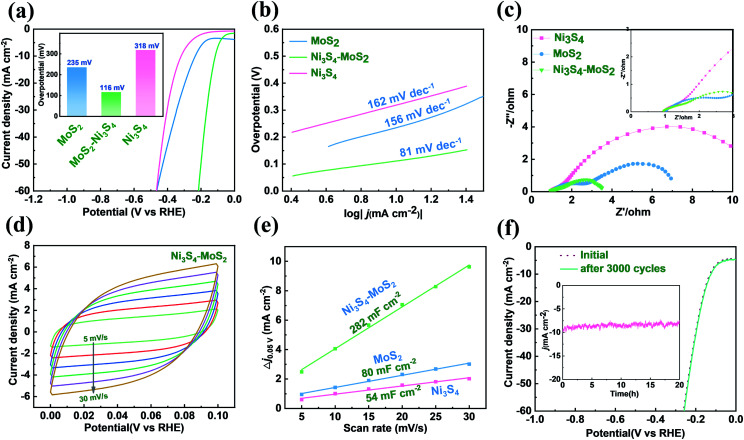
The HER behavior of Ni_3_S_4_–MoS_2_, Ni_3_S_4_ and MoS_2_ in 1 M KOH. (a) The LSV curves and overpotential (*η*_10_) without IR correction. (b) The Tafel slopes. (c) Nyquist plots collected at the overpotential of 180 mV. (d) CV curves of Ni_3_S_4_–MoS_2_ at the scan rates of 5, 10, 15, 20, 25 and 30 mV s^−1^, respectively. (e) Differences in current density variation (Δ*J* = *J*_a_ − *J*_c_) at 0.05 V *vs.* RHE plotted against scan rate fitted to linear regression for estimation of *C*_dl_ values of Ni_3_S_4_–MoS_2_, Ni_3_S_4_ and MoS_2_. (f) The initial and 3000th polarization curves of Ni_3_S_4_–MoS_2_. The inset is the chronoamperometric curve recorded at 10 mA for a continuous 20 h.

The HER activities of MoS_2_, Ni_3_S_4_–MoS_2_ and Ni_3_S_4_ on NF were measured in a 1 M KOH solution. There was a significant enhancement of the HER activity for Ni_3_S_4_–MoS_2_, as shown in Fig. 3a. The pure MoS_2_ exhibited an overpotential of 10 mA cm^−2^ (*η*_10_) at 235 mV, which is in agreement with the reported literature.^37^ The *η*_10_ of Ni_3_S_4_–MoS_2_ is 116 mV, which is much lower than that of the pure MoS_2_ (235 mV) and pristine Ni_3_S_4_ (318 mV). The heterostructure Ni_3_S_4_–MoS_2_ showed superior *η*_10_ in an alkaline solution and is comparable with the electrocatalysts reported in literature (see Table S4† for more details).^38–40^ The HER catalytic performance of the electrocatalysts with different Ni contents in Ni_3_S_4_–MoS_2_ was investigated, as shown in Fig. S3,† indicating that Ni_3_S_4_–MoS_2_ with 7.7 wt% Ni has the lowest overpotential (116 mV) at 10 mA cm^−2^ in the alkaline solution.

The Tafel curves of MoS_2_, Ni_3_S_4_–MoS_2_ and Ni_3_S_4_ on NF are shown in Fig. 3b. The Tafel slope of Ni_3_S_4_–MoS_2_ (81 mV dec^−1^) was much lower than that of MoS_2_ (156 mV dec^−1^), indicating that Ni_3_S_4_ played a key role in promoting the kinetics of HER. The EIS diagrams exhibited similar impedance characteristics, which implied similar electrochemical processes of these samples (Fig. 3c). Ni_3_S_4_–MoS_2_ showed a much lower charge-transfer-resistance (*R*_ct_) value when compared with the other catalysts, suggesting that Ni_3_S_4_–MoS_2_ had better charge-transfer property and HER kinetics. In addition, not only the electric conductivity but also the wettability of MoS_2_ was influenced by Ni_3_S_4_. The contact angle tests of the materials in 1 mol L^−1^ KOH electrolyte were carried out to explain such influence. The contact angle decreased from 21.36° for MoS_2_ to 15° for Ni_3_S_4_–MoS_2_ (Fig. S3†). It is shown that Ni_3_S_4_–MoS_2_ had better wettability in the KOH electrolyte than that of initial MoS_2_.

Electrochemical active surface area (ECSA) is a standard parameter applied in the evaluation of electrochemical catalysts. To investigate the exposed active sites, the ECSA of Ni_3_S_4_–MoS_2_, MoS_2_ and Ni_3_S_4_ were calculated by the double-layer capacitance (*C*_dl_) through plotting CV curves. The CV curves of the samples were tested in the potential range of 0.0–0.1 V at the scan rates of 5, 10, 15, 20, 25 and 30 mV s^−1^ in 1.0 M KOH, respectively (Fig. 3d and S5†). The *C*_dl_ value of Ni_3_S_4_–MoS_2_ was 282 mF cm^−2^, which is much higher than that of MoS_2_ (80 mF cm^−2^) and Ni_3_S_4_ (54 mF cm^−2^) (Fig. 3e), indicating that the additional electrochemical active sites were generated after Ni_3_S_4_ was introduced. The *C*_dl_ value of Ni_3_S_4_–MoS_2_ was much more than that of MoS_2_ and Ni_3_S_4_, indicating there are more active sites exposed for HER.

To evaluate the long-term stability of the heterostructure Ni_3_S_4_–MoS_2_, it was subjected to 3000 continuous CV cycles in an alkaline environment from 0 to −0.3 V *vs.* RHE. The LSV curves had no clear changes before and after 3000 CV cycles (Fig. 3f), indicating that Ni_3_S_4_–MoS_2_ has excellent catalytic stability during the electrochemical process. Besides, Ni_3_S_4_–MoS_2_ has a stable HER current at a constant current of 10 mA *versus* time over a 20 h period in 1 M KOH (Fig. 3f). Simultaneously, the morphology of Ni_3_S_4_–MoS_2_ was well preserved (Fig. S6†), demonstrating excellent catalytic stability during the alkaline HER process.

### Mechanism of Ni_3_S_4_–MoS_2_ for HER

**Fig. 4:**
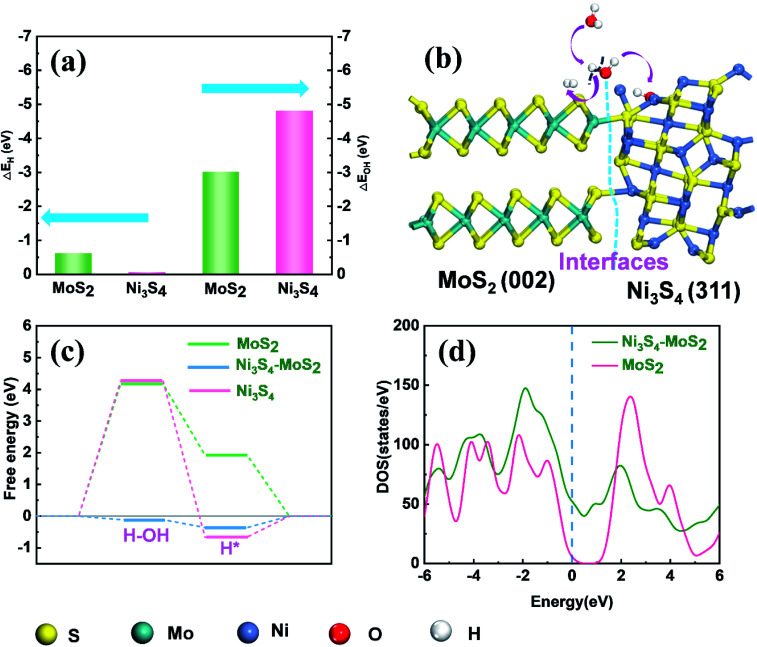
(a) DFT-calculated adsorption energies of H and OH at different sites on the surfaces of Ni_3_S_4_–MoS_2_, respectively. (b) The illustration of a mechanism for the electrocatalytic HER under alkaline conditions. (c) Free energy diagrams on the surface of MoS_2_, Ni_3_S_4_ and Ni_3_S_4_–MoS_2_ in alkaline solution. (d) DOSs of pristine MoS_2_ and Ni_3_S_4_–MoS_2_.

According to literature,^41,42^ DFT calculations were also carried out to gain insight into the underlying mechanism of Ni_3_S_4_–MoS_2_ towards the HER activity. In an alkaline medium, the HER reaction mainly includes three steps: water adsorbed on the catalyst, water dissociation, H* formation and H_2_ generation.^43,44^ The water dissociation step is considered as the important step for the HER catalytic property in an alkaline solution. The chemisorption free energies of OH (Δ*E*_OH_) and H (Δ*E*_H_) on the different sites of Ni_3_S_4_–MoS_2_ were calculated, respectively. To study the optimal chemisorption free energies (Δ*E*), several feasible positions were chosen for the adsorption of OH and H (Fig. S7†). The chemisorption free energy of H adsorbed on the (002) plane of MoS_2_ (Δ*E*_H_ = −0.81 eV) was lower than that absorbed on the (311) plane of Ni_3_S_4_ (Δ*E*_H_ = −0.027 eV), indicating that H was inclined to be adsorbed on the (002) plane of MoS_2_. Compared with the (002) plane of MoS_2_ (Δ*E*_OH_ = −3.0 eV), the (311) plane of Ni_3_S_4_ showed a predominant binding energy towards OH (Δ*E*_OH_ = −4.8 eV), which is attributed to the bonding ability between OH and Ni (Fig. 4a). Therefore, OH is intensely adsorbed on the (311) plane of Ni_3_S_4_ and H on the (002) plane of MoS_2_. The appropriate oxidation of Ni in Ni_3_S_4_–MoS_2_ contributes to the adsorption energy of OH. Simultaneously, the partial reduction of S in Ni_3_S_4_–MoS_2_ is beneficial for the adsorption of H. It demonstrated a synergistic effect of Ni_3_S_4_–MoS_2_ with chemisorption of H (on the (002) plane of MoS_2_) and OH (on the (311) plane of Ni_3_S_4_) accelerated the rate-determining water dissociation steps of HER.

The free energy diagrams on the surfaces of MoS_2_, Ni_3_S_4_ and Ni_3_S_4_–MoS_2_ are shown in Fig. S8.† The Δ*G*_H*_ of Ni_3_S_4_–MoS_2_ was −0.36 eV, which was much lower than that of pure MoS_2_ (1.92 eV), indicating the superior capacity of Ni_3_S_4_–MoS_2_ for H* adsorption (Table S4†), which benefited from the electron redistribution between Ni_3_S_4_ and MoS_2_. For pure MoS_2_, the free energy barrier of water dissociation Δ*G*_H_2_O_ is as high as 4.2 eV, which distinctly hindered the dissociation of H_2_O to H*. Moreover, the Δ*G*_H2O_ of Ni_3_S_4_–MoS_2_ was only −0.10 eV, which was much lower than that of MoS_2_ (4.2 eV) and Ni_3_S_4_ (4.3 eV). It is indicated that the Δ*G*_H_2_O_ of Ni_3_S_4_–MoS_2_ efficiently decreased because of the existence of a heterostructure. Hence, the HER process on Ni_3_S_4_–MoS_2_ is highly accelerated and in accordance with our experimental results.

Moreover, the heterostructure improved the electrical transport efficiency of Ni_3_S_4_–MoS_2_. It is found that the total density of state (DOS) curve of MoS_2_ shows a clear band gap at the region around 0 eV, confirming the typical semiconductor characteristic. The peak of the valence band of the heterostructure Ni_3_S_4_–MoS_2_ is close to 0 eV (Fig. 4d), leading to the enhanced excitation of charge carriers to the conduction band and showing better electric conductivity, which is consistent with the EIS tests.

## Conclusions

In summary, we fabricated the sphere-shaped heterostructure Ni_3_S_4_–MoS_2_ by a one-pot hydrothermal method. The as-synthesized catalyst with the activated interfaces generated abundant active sites and improved the electrical transport efficiency. Benefiting from engineering the heterostructure, the Ni_3_S_4_–MoS_2_ demonstrated the low overpotential of 116 mV with the corresponding Tafel slope of 81 mV dec^−1^ and long-term stability of over 20 h. DFT calculations proved that the heterostructure Ni_3_S_4_–MoS_2_ resulted in electron redistribution, which indicated the presence of a synergistic effect with MoS_2_ as the hydrogen acceptor and Ni_3_S_4_ as the hydroxyl acceptor, and effectively reduced the intermediate energy barrier of the water dissociation. Hence showing outstanding HER performance in alkaline solution. This work opens the door to develop low-cost and high-activity HER electrocatalyst Ni_3_S_4_–MoS_2_*via* heterostructure engineering.

## Conflicts of interest

The authors declare no competing financial interest.

## Supplementary Material

RA-011-D1RA02828F-s001
