# Supplementary material for: Heterostructure Ni3S4–MoS2 with interfacial electron redistribution used for enhancing hydrogen evolution
Source: RSC Adv. 2021 Jun 1;11(32):19630–8. doi: 10.1039/d1ra02828f (PMC9033570; doi:10.1039/d1ra02828f)
Supplement: RA-011-D1RA02828F-s001 [file RA-011-D1RA02828F-s001.pdf]

Electronic Supplementary Material (ESI) for RSC Advances.
This journal is © The Royal Society of Chemistry 2021

Supporting Information

Heterostructure Ni_3S_4-MoS_2 with interfacial electron redistribution used for enhancing hydrogen evolution

Jingmin Ge,[a] Jiaxing Jin,[a] Yanming Cao,[a] Meihong Jiang,[a] Fazhi Zhang,[a] Hongling Guo,*[b] Xiaodong Lei*[a]

[a] State Key Laboratory of Chemical Resource Engineering, Beijing University of Chemical Technology, Beijing 100029, China. Tel: +86-10-64455357;

[b] Institute of Forensic Science, Ministry of Public Security, Beijing 100038, China

*Corresponding author

E-mail: guohongling1234@163.com; leixd@mail.buct.edu.cn

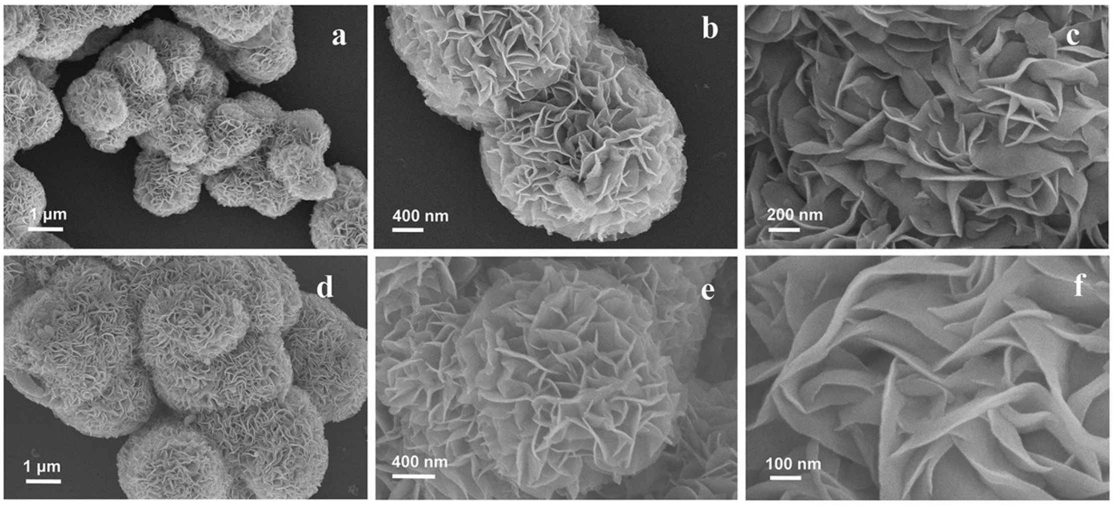

Fig. S1. (a-c) SEM images of pure MoS_2. (d-e) SEM images of Ni_3S_4-MoS_2.

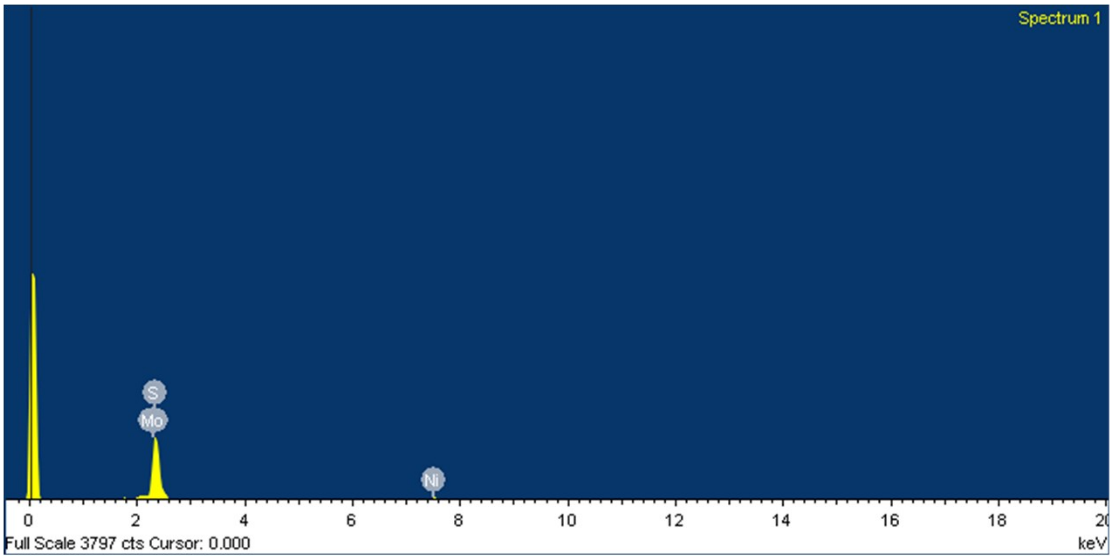

Fig. S2. EDS spectrum of Ni_3S_4-MoS_2.

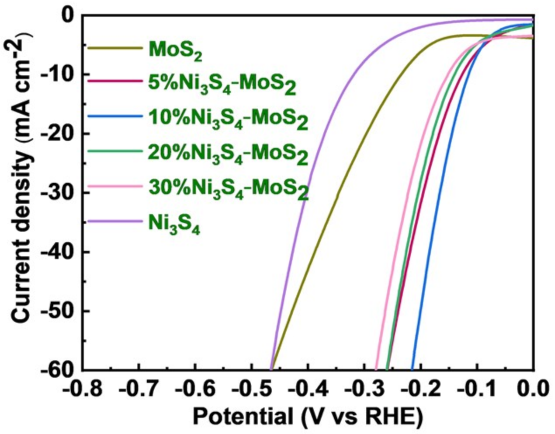

Fig. S3. LSV curves of Ni_3S_4-MoS_2 with different Ni contents in 1M KOH.

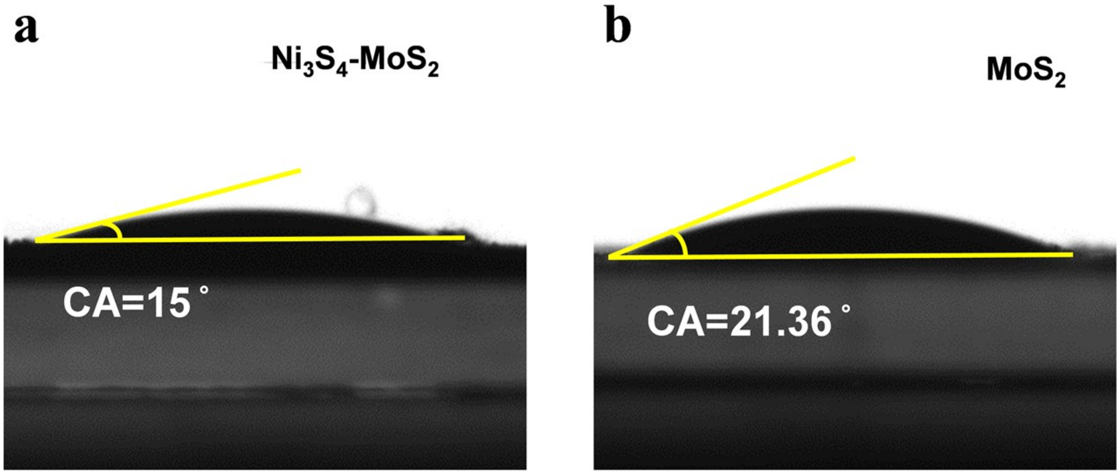

Fig. S4. Contact angles of Ni_3S_4-MoS_2 (a) and MoS_2 (b).

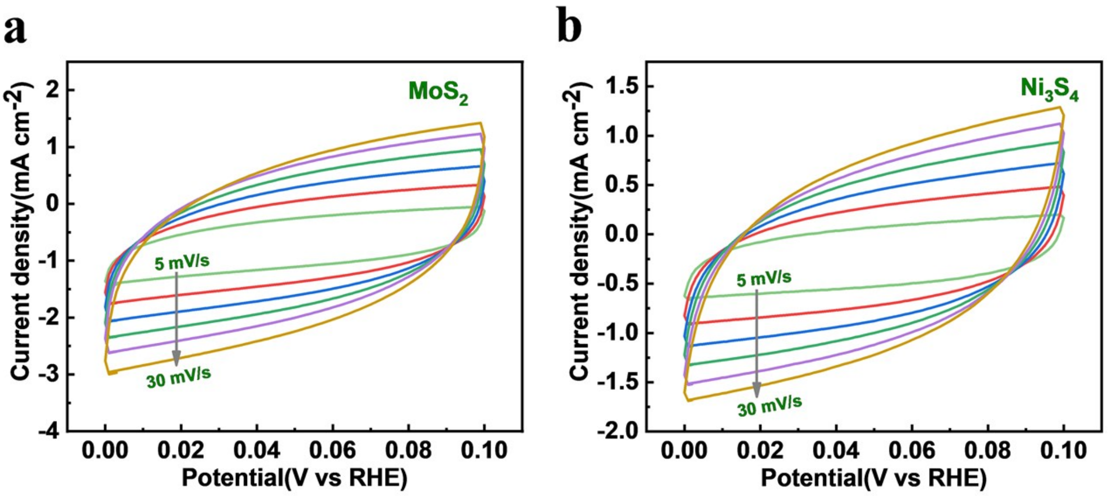

Fig. S5. CV curves of MoS_2 (a) and Ni_3S_4 (b) at the scan rates of 5, 10, 15, 20, 25 and 30 mV s^{-1} in 1.0 M KOH.

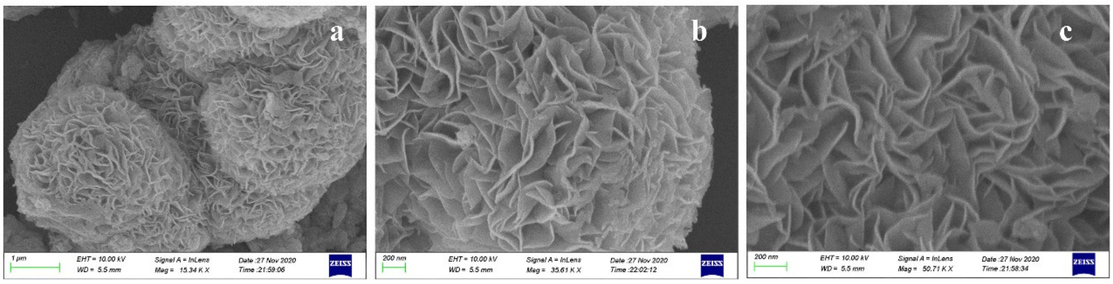

Fig. S6. (a and b) SEM images of Ni_3S_4-MoS_2 after 3000 CV curves. (c) SEM image of Ni_3S_4-MoS_2 after a stability test for 20 h.

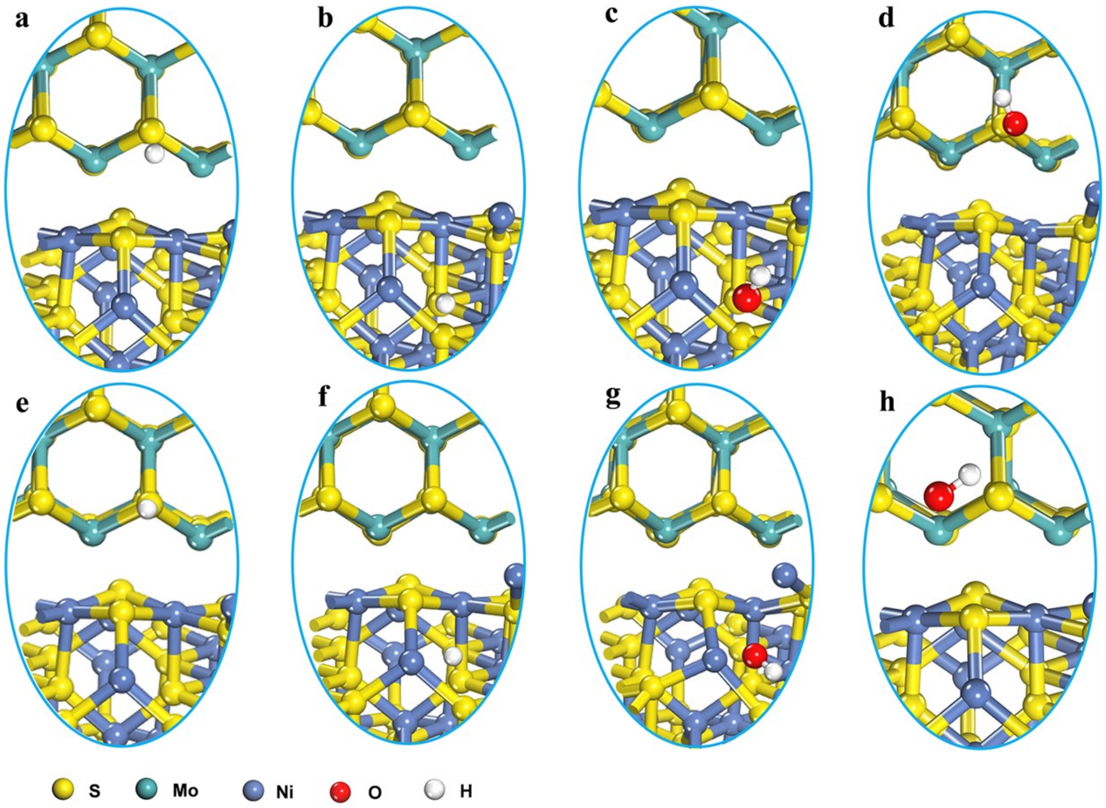

Fig. S7. Top-view of the initial structures for H adsorption on the (002) plane of MoS_2 (a) and the (311) plane of Ni_3S_4 (b), and for OH adsorption on the (311) plane of Ni_3S_4 (c) and the (002) plane of MoS_2 (d) in the heterostructure Ni_3S_4-MoS_2. The optimized structures for H adsorption on the (002) plane of MoS_2 (e) and the (311) plane of Ni_3S_4 (f), and for OH adsorption on the (311) plane of Ni_3S_4 (g) and the (002) plane of MoS_2 (h) in the heterostructure Ni_3S_4-MoS_2.

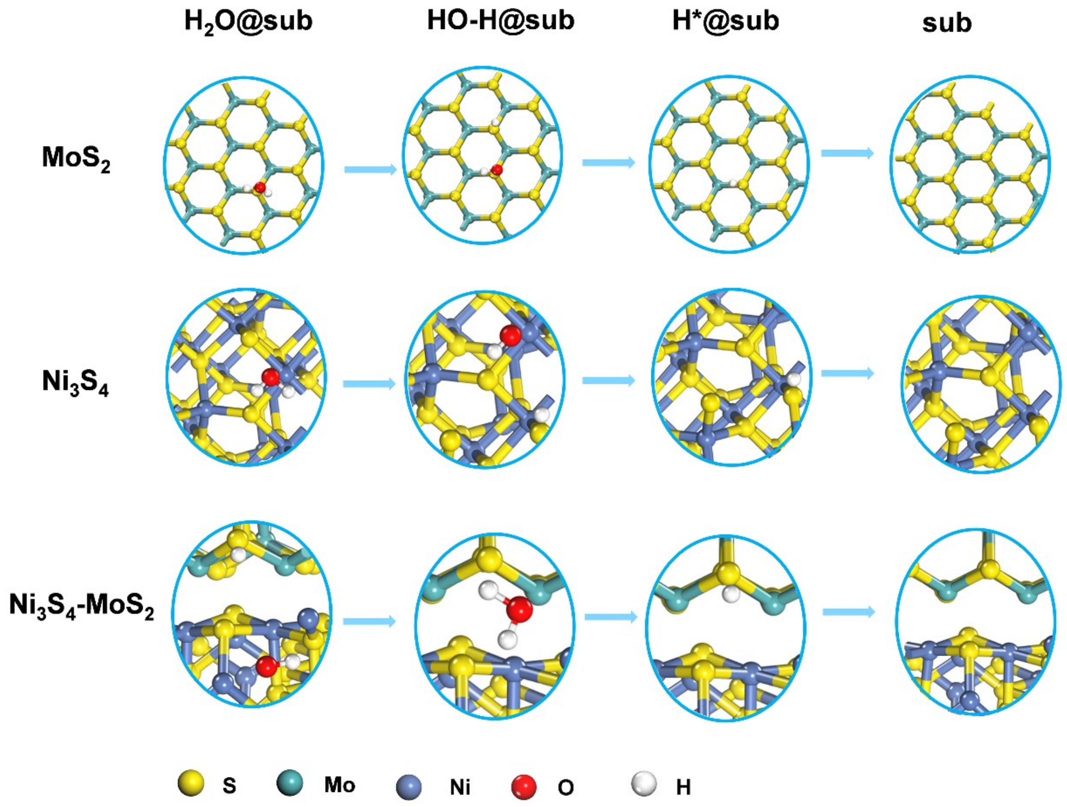

Fig. S8. HER reaction pathways and relevant structures of the most possible intermediate steps on the surface of MoS_2, Ni_3S_4 and Ni_3S_4-MoS_2, respectively.

Table S1 Ni_4S_3-MoS_2 samples with different Ni contents.

**Crystalline**	**ICP-OES test results**
5%Ni_4S_3-MoS_2	Ni 4.37 wt.%
10%Ni_4S_3-MoS_2	Ni 7.7 wt.%
20%Ni_4S_3-MoS_2	Ni 14.2 wt.%
30%Ni_4S_3-MoS_2	Ni 20.7 wt.%

Table S2 The EDS quantitative analysis results of Ni_3S_4-MoS_2.

Element	Weight%	Atomic%
S	55.75	77.26
Ni	7.63	5.77
Mo	36.63	16.96
Totals	100.00	

Table S3 Bader charge analysis of Ni_3S_4-MoS_2.

Element	Bader Charge /|**e**|
Mo-10	0.05
Mo-20	0.02
Ni-5	-0.13
Ni-8	-0.27
Ni-11	-0.14
Ni-17	-0.06
Ni-22	-0.05
S-15	0.21
S-35	0.19
S-41	0.16
S-45	0.15

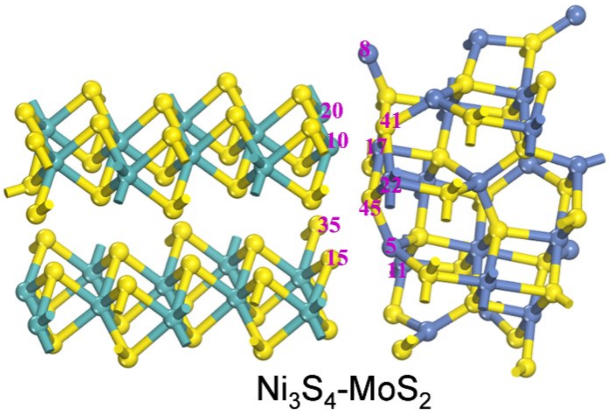

Ni_3S_4-MoS_2

Table S4 The adsorption free energy (ΔE) and Gibbs free energy (ΔG) of hydrogen generations and water dissociation during HER on the surface of MoS_2, Ni_3S_4 and Ni_3S_4-MoS_2.

	ΔE(H*)/eV	ΔG(H*)/eV	ΔE (H_2O)/eV	ΔG (H_2O)/eV
MoS_2	1.69	1.93	3.73	4.18
Ni_3S_4	-0.90	-0.66	3.84	4.29
Ni_3S_4-MoS_2	-0.60	-0.36	-0.55	-0.10

Table S5 Summary of several representative recently reported HER electrocatalysts employed in acidic and alkaline electrolytes.

Catalyst	Electrolyte	η_{10} (mV)	Tafel slope (mV dec^{-1})	Reference
NiS_2/MoS_2	1M KOH	235	58	[1]
MoO_3-MoS_2	0.5 M H_2SO_4	200	74	[2]
MoP/MoS_2	1M KOH	92	59.8	[3]
Ni_2P/MoS_2/N:CNT	1M KOH<br>0.5 M H_2SO_4	149<br>39.5	60.22<br>57.8	[4]
Mo_2N–Mo_2C/HGr	0.5 M H_2SO_4<br>1M KOH	154<br>157	55<br>68	[5]
MoSSe@rGO	0.5 M H_2SO_4	135(η_5)	51	[6]
(CoMo)S_2/graphene	0.5 M H_2SO_4	100	60.8	[7]
Fe-MoS_2/Ni_3S_2/NF	1M KOH	130.6	112.7	[8]
Co_3O_4/MoS_2	1M KOH	205	98	[9]
MoS_2/Ni_3S_2	1M KOH	110	83.1	[10]
MoS_2-Ni_3S_2	1M KOH	98	61	[11]
$Ni(OH)_2/MoS_2$	1M KOH	156	56.4	[12]
MoS_2|NiS|MoO_3	1M KOH	95	54.5	[13]
Ni-MoS_2/CC	1M NaOH	107	162	[14]

Ni–MoS_2/NCNTs	0.5 M H_2SO_4<br>1M KOH	158<br>179	69.3<br>62.3	[15]
MoS_2/rGO	0.5 M H_2SO_4	160	52	[16]
MoS_2/g-CN	0.5 M H_2SO_4	141	57	[17]
NC@MoS_2/Ni-NC	1M KOH	96.3	81.1	[18]
CoS_2-MoS_2	1M KOH	130	66.8	[19]
Ni_3S_4-MoS_2	1M KOH	116	81	***This work***

References

1. P. Y. Kuang, T. Tong, K. Fan and J. G. Yu, In Situ Fabrication of Ni-Mo Bimetal Sulfide Hybrid as an Efficient Electrocatalyst for Hydrogen Evolution over a Wide pH Range, *Acs Catalysis*, 2017, **7**, 6179-6187.
2. X. Hou, A. Mensah, M. Zhao, Y. Cai and Q. Wei, Facile controlled synthesis of monodispersed MoO_3-MoS_2 hybrid nanospheres for efficient hydrogen evolution reaction, *Applied Surface Science*, 2020, **529**, 147115.
3. Q. Liu, Z. Xue, B. Jia, Q. Liu, K. Liu, Y. Lin, M. Liu, Y. Li and G. Li, Hierarchical Nanorods of MoS_2/MoP Heterojunction for Efficient Electrocatalytic Hydrogen Evolution Reaction, *Small*, 2020, **16**, e2002482.
4. M. Kim, M. A. R. Anjum, M. Lee, B. J. Lee and J. S. Lee, Activating MoS_2 Basal Plane with Ni_2P Nanoparticles for Pt-Like Hydrogen Evolution Reaction in Acidic Media, *Advanced Functional Materials*, 2019, **29**, 1809151.
5. H. Yan, Y. Xie, Y. Jiao, A. Wu, C. Tian, X. Zhang, L. Wang and H. Fu, Holey Reduced Graphene Oxide Coupled with an Mo_2N-Mo_2C Heterojunction for Efficient Hydrogen Evolution, *Adv Mater*, 2018, **30**, 1704156.
6. B. Konkena, J. Masa, W. Xia, M. Muhler and W. Schuhmann, MoSSe@reduced graphene oxide nanocomposite heterostructures as efficient and stable electrocatalysts for the hydrogen evolution reaction, *Nano Energy*, 2016, **29**, 46-53.
7. L. X. Chen, Z. W. Chen, Y. Zhang, C. C. Yang and Q. Jiang, Insight into the excellent catalytic activity of $(CoMo)S_2$/graphene for hydrogen evolution reaction,

Applied Catalysis B: Environmental, 2019, **258**, 118012.

8. J. Y. Xue, F. L. Li, Z. Y. Zhao, C. Li, C. Y. Ni, H. W. Gu, P. Braunstein, X. Q. Huang and J. P. Lang, A hierarchically-assembled Fe-MoS_2/Ni_3S_2/nickel foam electrocatalyst for efficient water splitting, *Dalton Transactions*, 2019, **48**, 12186-12192.

9. A. Muthurasu, V. Maruthapandian and H. Y. Kim, Metal-organic framework derived Co_3O_4/MoS_2 heterostructure for efficient bifunctional electrocatalysts for oxygen evolution reaction and hydrogen evolution reaction, *Applied Catalysis B-Environmental*, 2019, **248**, 202-210.

10. J. Zhang, T. Wang, D. Pohl, B. Rellinghaus, R. H. Dong, S. H. Liu, X. D. Zhuang and X. L. Feng, Interface Engineering of MoS_2/Ni_3S_2 Heterostructures for Highly Enhanced Electrochemical Overall-Water-Splitting Activity, *Angewandte Chemie-International Edition*, 2016, **55**, 6702-6707.

11. Y. Q. Yang, K. Zhang, H. L. Ling, X. Li, H. C. Chan, L. C. Yang and Q. S. Gao, MoS_2-Ni_3S_2 Heteronanorods as Efficient and Stable Bifunctional Electrocatalysts for Overall Water Splitting, *Acs Catalysis*, 2017, **7**, 2357-2366.

12. Z. Y. He, Q. Y. Liu, Y. M. Zhu, T. Tan, L. X. Cao, S. J. Zhao and Y. Chen, Defect-Mediated Adsorption of Metal Ions for Constructing Ni Hydroxide/MoS2 Heterostructures as High-Performance Water-Splitting Electrocatalysts, *Acs Applied Energy Materials*, 2020, **3**, 7039-7047.

13. C. Wang, B. Tian, M. Wu and J. Wang, Revelation of the Excellent Intrinsic Activity of MoS_2|NiS|MoO_3 Nanowires for Hydrogen Evolution Reaction in

Alkaline Medium, *ACS Applied Materials & Interfaces*, 2017, **9**, 7084-7090.

14. Y. Li, L. X. Wang, A. L. Song, M. R. Xia, Z. P. Li and G. J. Shao, The study on the active origin of electrocatalytic water splitting using Ni-MoS_2 as example, *Electrochimica Acta*, 2018, **268**, 268-275.
15. T. Dong, X. Zhang, P. Wang, H.-S. Chen and P. Yang, Formation of Ni-doped MoS_2 nanosheets on N-doped carbon nanotubes towards superior hydrogen evolution, *Electrochimica Acta*, 2020, **338**, 135885.
16. Y. Wang, F. Lu, K. Su, N. Zhang, Y. Zhang, M. Wang and X. Wang, Engineering Mo-O-C interface in MoS_2@rGO via charge transfer boosts hydrogen evolution, *Chemical Engineering Journal*, 2020, **399**, 126018.
17. M. Q. Guan, C. Wang, S. Li, H. W. Du and Y. P. Yuan, Understanding the Enhanced Electrocatalytic Hydrogen Evolution via Integrating Electrochemically Inactive g-C_3N_4: The Effect of Interfacial Engineering, *Acs Sustainable Chemistry & Engineering*, 2020, **8**, 10313-10320.
18. Q. Luo, C. Wang, H. Xin, Y. Qi, Y. Zhao, J. Sun and F. Ma, Hollow Sandwiched Structure of Ni-Modified MoS_2 Wrapped into Symmetrical N-Doped Carbon toward a Superior Hydrogen Evolution Electrocatalyst, *ACS Sustainable Chemistry & Engineering*, 2021, **9**, 732-742.
19. Y. X. Zhou, W. Zhang, M. H. Zhang, X. X. Shen, Z. X. Zhang, X. D. Meng, X. S. Shen, X. H. Zeng and M. Zhou, Hetero-structured CoS_2-MoS_2 hollow microspheres with robust catalytic activity for alkaline hydrogen evolution, *Applied Surface Science*, 2020, **527**, 146847.
